# Characterizing Prescription Stimulant Use in Adults with Overdose Involving Psychostimulants

**DOI:** 10.18103/mra.v13i7.6741

**Published:** 2025-07

**Authors:** Elizabeth Bolt, Wen-Jan Tuan

**Affiliations:** 1 Pennsylvania State University College of Medicine, Hershey, PA; 2 Departments of Family and Community Medicine and Public Health Science, Pennsylvania State University College of Medicine, Hershey, PA

## Abstract

The objective of this brief report is to characterize the use of prescription stimulants in adults with psychostimulant-related overdose in the United States amid the rising prevalence of stimulant prescriptions and stimulant-related overdose deaths. Using the International Classification of Diseases, Tenth Revision coding system, we identified adults with diagnoses related to overdose involving psychostimulants between 2010 and 2022 and used descriptive statistics to characterize these individuals. The analysis included 15,744 adults with stimulant-related overdoses (16.5% intentional, 83.5% unintentional). We found that 17.7% of adults with stimulant-related overdose events had a previous stimulant prescription. Nearly half of those with a stimulant-related overdose had a previously identified mental health disorder (49.9%) or substance use disorder (43.2%). The prevalence of overdose in high-risk adult populations highlights the need for additional safety interventions. Risk-benefit analysis and tailored guidelines for prescribing stimulants, especially in populations with mental health or substance use disorders, are warranted to mitigate overdose risk and enhance patient safety.

## Introduction

Over the past two decades, drug overdose deaths have surpassed one million in the United States.^1,2^ A dramatic increase in prescription opioids were an unfortunate catalyst for the first wave of the opioid epidemic; now in its fourth wave, stimulants are a primary driver of overdoses across the United States.^3^ Similar to the rise witnessed in opioid prescriptions, prescriptions for stimulants have doubled since 2011.^4^ Overdose deaths involving stimulants have risen tenfold over the last decade in the United States,^5^ from 2,635 deaths related to psychostimulants in 2012 to 34,022 psychostimulant-related deaths in 2022.^1^ While stimulant medications are mainly used to treat attention deficit hyperactivity disorder (ADHD) in children and adolescents, more than 55% of total prescription stimulants have been dispensed to adults since 2015.^6^ Stimulants continue to be prescribed broadly in populations with limited safety data, such as adults, even though one-third of adults prescribed stimulants report misuse.^7^ Between 2006 and 2011, non-medical use of prescription stimulants increased 67.1% in adults,^8^ and by 2019, stimulants accounted for nearly 23% of all drug overdose deaths.^9^ Despite these clear trends, the role of prescription stimulants in the overdose epidemic remains unclear. The objective of this brief report is to characterize prescription stimulant use in adults with a history of psychostimulant-related overdose in the United States.

## Methods

This retrospective study utilized the TriNetX Research Network database,^10^ which contains de-identified electronic health records (EHRs) from 55 healthcare organizations. The study population consisted of adults (≥ 18 years) with diagnoses related to intentional and unintentional overdose involving psychostimulants (amphetamines, methylphenidate, and methamphetamine) between January 1, 2010 and December 31, 2022. This time period was intentionally selected to include stimulant use data during the height of the COVID-19 pandemic. Overdose events were captured with the International Classification of Diseases, Tenth Revision (ICD-10) diagnosis codes. For individuals with multiple overdose events, only data from the first overdose event was included in the analysis. Individual characteristics consisted of age at overdose, sex, race/ethnicity, mental health diagnoses (both lifetime and in the six months leading up to the overdose), lifetime substance use disorder (SUD) diagnoses, and SUD diagnoses in the six months prior to overdose.

We assessed between group differences in opioid use disorder (OUD) and stimulant use disorder (StUD) both independently and grouped with other SUDs. We also evaluated adults who received stimulant prescriptions one month and six months prior to overdose. Stimulant prescriptions were identified using RxNorm medication codes based on generic and brand names. We conducted descriptive statistical analyses to characterize individuals who experienced overdose events, examining key demographic and clinical attributes. To evaluate population-level differences between intentional and unintentional overdoses, we employed the Mann-Whitney U test for continuous variables (given the potential for non-normal distributions) and the chi-square test for categorical variables. These comparative analyses allowed us to identify significant distinctions in characteristics across overdose intent categories. Detailed information regarding diagnosis and medication codes used in this study is provided in Supplemental Table S.1.

## Results

In total, there were 15,744 adults that experienced a stimulant-related overdose event (16.5% intentional and 83.5% unintentional). The mean age for individuals with a stimulant-related overdose was 38.5 years, and the majority of overdose events involved individuals classified as white (69%) and male (65.4%). Almost half of all overdoses involved individuals with a diagnosed mental health disorder (49.9%) or substance use disorder (43.2%). A previous stimulant prescription was recorded in nearly one-fifth of these cases (17.7%), and 11.2% of overdoses involved a stimulant prescription within one month of the overdose event.

Individuals with intentional overdose (n = 2,592) were more often female (45.1% vs 32.5%), white (72.8% vs 68.2%), and had a higher prevalence of mental health disorder diagnoses compared to those with unintentional overdose (n = 13,152). They also had a higher prevalence of stimulant prescriptions—both over their lifetime (29.1% vs 15.4%) and in the one- and six-month periods prior to the overdose. In contrast, those with unintentional overdose had a higher prevalence of lifetime SUD diagnoses (44% vs 39.1%) and SUD diagnoses in the six months preceding overdose (28.1% vs 25.7%) compared to those with intentional overdose. Furthermore, they had a higher prevalence of diagnosed StUD both over their lifetime (21.5% vs 16.6%) and within six months of overdose (13.7% vs 10.5%). Detailed information is provided in Table 1.

## Discussion

Although the majority of psychostimulant-related overdose events did not occur among adults prescribed stimulants during this time period, stimulant prescriptions can contribute to significant morbidity and mortality.^1,5^ We found that adults with intentional overdose were more likely to have a previous mental health diagnosis, while those with unintentional overdose were more likely to have a prior SUD or StUD diagnosis. The high prevalence of overdose events among those with previously identified mental health disorders and SUDs suggests opportunity to enhance safety when prescribing stimulants. Although stimulants are a relatively safe medication when used within the scope of their intent, this data suggests that access to prescription stimulants for adults in high-risk groups must be a consideration to prevent adverse outcomes such as overdose.

While the benefits of these medications for the treatment of ADHD in healthy pediatric populations most often outweigh the risks, stimulant prescriptions in adults have not been thoroughly studied. Concerns have been raised that the chronic use of stimulants may lead to the development of addiction, as those with ADHD and comorbid SUD have a greater risk of poly-substance use,^11^ increasing their likelihood of overdose. Our study found that when compared with unintentional stimulant-related overdose, a larger proportion of adults with intentional stimulant-related overdose were found to have previous stimulant prescriptions. Additionally, a greater proportion of those with intentional overdose had been dispensed stimulants in the one- or six-month timeframe leading up to the overdose event. These findings highlight the urgent need to develop more effective tools to assist healthcare professionals with monitoring prescription stimulant use, especially regarding access to stimulants in those with mental health diagnoses or SUDs.

Stimulants have been shown to be effective in treating certain mental health conditions such as ADHD in those with concomitant SUD, especially at higher doses, posing a greater risk of misuse and diversion of these medications.^12^ Moreover, other mental health disorders (i.e. anxiety, bipolar disorder, and SUDs) can masquerade as ADHD in adults,^13^ highlighting the need for additional guidance to appropriately characterize the risks and benefits of stimulant use in this heterogenous population. Despite the fact that CDC guidelines and opioid risk tools have influenced prescribing patterns for opioids,^14^ similar guidance does not exist for stimulants. After identifying high-risk adult groups that may benefit from an alternative approach to care for symptoms of ADHD,^15^ we must now develop and implement screening measures as well as high quality risk-benefit analysis tools to better inform prescribing practices. These data are a foundational step in that endeavor.

While we believe these data are important to disseminate, there are key limitations that should be considered. Most notably, these data were sourced from EHRs and relied on ICD-10 codes for analysis. These have well documented imperfections,^16^ including low sensitivity of detecting illicit substance use.^17^ Moreover, the ICD-10 codes utilized may not encompass all stimulant-related overdose events, therefore, the magnitude of overdoses associated with stimulant prescriptions may be underestimated by this study. Additionally, we only included an individual’s first overdose event in the analysis, so the data do not reflect those that experienced multiple overdoses. Finally, while the time interval of the study encompasses stimulant use data during the COVID-19 pandemic, we are unable to address potential changes in prescription stimulant use in the post-pandemic era.

## Conclusion

To this date, our knowledge about the long-term impact of prescription stimulants is limited. We do not currently have prescription guidelines to inform long-term management of stimulant prescriptions, nor to mitigate the risk of psychostimulant-related overdose. We believe these data are an important early step in providing critical information to medical providers and to advance safety for patients prescribed stimulants. Additional research is required to further inform the risks and benefits of prescription stimulants across the lifespan and to prevent morbidity and mortality associated with these medications.

## Supplementary Material

1

## Figures and Tables

**Table 1:** Summary Statistics of Adults with Stimulant-Related Overdose

		Intentional versus Unintentional Overdose		
	N = 15,744	Intentional (n = 2,592)	Unintentional (n = 13,152)	P-value
Age at Overdose, mean	38.5	33.5	39.5	
Female	5,440 (34.6)	1,168 (45.1)	4,272 (32.5)	<0.001
Race				
White	10,862 (69)	1,888 (72.8)	8,974 (68.2)	<0.001
Black	1,892 (12)	285 (11)	1,607 (12.2)	0.08
Asian	165 (1)	32 (1.2)	133 (1)	0.31
Other	2,825 (17.9)	387 (2.3)	327 (2.5)	<0.001
Ethnicity				
Hispanic	1,118 (7.1)	190 (7.3)	928 (7.1)	0.62
Mental Health Diagnosis, lifetime*	7,853 (49.9)	1,346 (51.9)	6,507 (49.5)	0.02
Mental Health Diagnosis, 6 months prior to overdose*	5,236 (33.3)	931 (35.9)	4,305 (32.7)	<0.01
SUD Diagnosis, lifetime				
SUD†	6,800 (43.2)	1,014 (39.1)	5,786 (44)	<0.001
OUD	1,970 (12.5)	295 (11.4)	1,675 (12.7)	0.06
StUD	3,262 (20.7)	429 (16.6)	2,833 (21.5)	<0.001
SUD Diagnosis, 6 months prior to overdose				
SUD†	4,363 (27.7)	665 (25.7)	3,698 (28.1)	0.01
OUD	1,050 (6.7)	175 (6.8)	875 (6.7)	0.85
StUD	2,075 (13.2)	273 (10.5)	1,802 (13.7)	<0.001
Previous Stimulant Prescription, lifetime	2,785 (17.7)	755 (29.1)	2,030 (15.4)	<0.001
Previous Stimulant Prescription, 1 month prior to overdose	1,758 (11.2)	498 (19.2)	1,260 (9.6)	<0.001
Previous Stimulant Prescription, 6 months prior to overdose	2,066 (13.1)	567 (21.9)	1,499 (11.4)	<0.001

* Mental health diagnoses include anxiety, depression, attention-deficit/hyperactivity disorder, substance use disorder, and other/not specified.

† Any SUD, including those involving alcohol, opioids, cannabis, sedatives, cocaine, psychostimulants, nicotine, hallucinogens, inhalants or other psychoactive drugs.

SUD = Substance use disorder; OUD = Opioid use disorder; StUD = Stimulant use disorder
