# Supplementary material for: Characterizing Prescription Stimulant Use in Adults with Overdose Involving Psychostimulants
Source: Med Res Arch. Author manuscript; Available in PMC 2026 Jun 27. (PMC13309182; doi:10.18103/mra.v13i7.6741)
Supplement: 1 [file NIHMS2186602-supplement-1.pdf]

Supplemental Materials

Table S.1. Description of Coding Systems and Codes

Coding System	Code	Description
Overdose involving psychostimulants		
ICD-10*	T43621, T43631, T43651	Unintentional overdose
ICD-10	T43622, T43632, T43652	Intentional overdose
Prescription stimulants		
RxNorm	725, 3288, 6816, 6901, 352372, 700810	Stimulant medication
Baseline characteristics		
ICD-10	F01-F99	Mental health disorders
ICD-10	F11	Opioid use disorder
ICD-10	F15	Stimulant use disorder
ICD-10	F10-F19	Substance use disorders involving alcohol, opioids, cannabis, sedatives, cocaine, psychostimulants, nicotine, hallucinogens, inhalants or other psychoactive drugs

*TriNetX Research Network database curates crosswalk between ICD-9 and ICD-10 diagnoses, allowing users to exclusively rely on ICD-10 codes for data extraction.
